# P16-EXPRESSING SENESCENT CELLS ARE A DOUBLE-EDGED SWORD IN SHAPING IMMUNE RESPONSES WITH AGE

**DOI:** 10.1093/geroni/igac059.2940

**Published:** 2022-12-20

**Authors:** Blake Torrance, Hunter Panier, Andreia Cadar, Dominique Martin, Erica Lorenzo, Jenna Bartley, Laura Haynes

**Affiliations:** UConn Health, Farmington, Connecticut, United States; UConn Health, Farmington, Connecticut, United States; UConn Health, Farmington, Connecticut, United States; UConn Health, Farmington, Connecticut, United States; UConn Health, Farmington, Connecticut, United States; UConn Health, Farmington, Connecticut, United States; UConn Health, Farmington, Connecticut, United States

## Abstract

Aging results in the accumulation of senescent cells which can cause dysfunction in many contexts but the effects on immune responses remain unclear. Here, we aimed to probe the effects of clearing senescent cells in aged mice on the immune response to influenza infection. We utilized a powerful p16 trimodality reporter mouse model (p16-3MR): under the control of the p16 promoter, these mice express cassettes encoding luciferase, RFP, and herpesvirus thymidine kinase (HSV-TK). p16 is commonly upregulated in senescent cells so this model allows us to selectively delete those cells by treating with ganciclovir (GCV), which will induce apoptosis in cells expressing HSV-TK. We hypothesized that while p16-expressing senescent cells may exacerbate dysfunctional responses to a primary infection, they may play a protective role in resolving inflammation and fostering memory cell generation. We found that deletion of p16-expressing cells enhanced viral clearance and decreased infiltration of pro-inflammatory flu-specific CD8 T cells during the primary response to infection. Conversely, at 30 days post infection, there were fewer flu-specific CD8 memory T cells and lower amounts of anti-viral antibodies in the lungs of GCV treated mice. We also observed perturbations in memory T cell trafficking in GCV treated mice. Furthermore, GCV treated mice were unable to mount an effective memory response and were unable to control viral load following a heterosubtypic challenge. This suggests that targeting senescent cells may potentiate primary responses while limiting the ability to form durable and protective immune memory with age.

